# A missense variant in *DGKG* as a recessive functional variant for hepatic fibrinogen storage disease in Wagyu cattle

**DOI:** 10.1111/jvim.16865

**Published:** 2023-09-08

**Authors:** Joana G. P. Jacinto, Peter Wohlsein, Irene M. Häfliger, Michael Karl, Michael Pohlers, Lutz Plobner, Walter Grünberg, Cord Drögemüller

**Affiliations:** ^1^ Department of Veterinary Medical Sciences University of Bologna Bologna Switzerland; ^2^ Institute of Genetics, Vetsuisse Faculty University of Bern Bern Switzerland; ^3^ Department of Pathology University of Veterinary Medicine Hannover Hannover Germany; ^4^ Agrobiogen GmbH Hilgertshausen Germany; ^5^ Clinic for Cattle, University of Veterinary Medicine Hannover Hannover Germany; ^6^ Clinic for Ruminants and Herd Health Management, Justus‐Liebig University Giessen Gießen Germany

**Keywords:** *Bos taurus*, development, precision medicine, rare disease, storage disease, WGS

## Abstract

Hepatic fibrinogen storage disease (HFSD) was diagnosed in a 5‐month‐old Wagyu calf with a history of recurrent respiratory disease. It was characterized by lethargy, dehydration, acidemia, and increased liver enzyme activities. Histologically, disseminated hepatocytes were swollen and showed a single, sharply demarcated, faintly eosinophilic cytoplasmic inclusion with a ground‐glass appearance, with the nucleus in an eccentric position. Cytoplasmic inclusions did not stain with the periodic acid‐Schiff (PAS) reaction. Using a rabbit polyclonal antibody against fibrinogen, the cytoplasmic vacuoles in the hepatocytes stained intensely. Electron microscopy disclosed hepatocytes with membrane‐bound cytoplasmic inclusions filled with fine granular material interspersed with a few coarse‐grained electron‐dense granules. A trio whole‐genome sequencing approach identified a deleterious homozygous missense variant in *DGKG* (p.Thr721Ile). The allele frequency in 209 genotyped Wagyu was 7.2%. This is a report of a *DGKG*‐related recessive inherited disorder in cattle and adds *DGKG* to the list of candidate genes for HFSD in other species.

**Abbreviations**
ABC: avidin‐biotin peroxidase complex
AST: aspartate aminotransferase
CK: creatine kinase
DGK: diacylglycerol kinase
DGKG: diacylglycerol kinase gamma protein
DPAS: PAS with diastase
GGT: gamma‐glutamyl transferase
GLDH: glutamate dehydrogenase
HE: hematoxylin and eosin
HFSD: hepatic fibrinogen storage disease
IGV: Integrative Genomics Viewer
PAS: periodic acid‐Schiff
RBC: red blood cells
WGS: whole‐genome sequencing

## 1 INTRODUCTION

Hepatic fibrinogen storage disease (HFSD) is an endoplasmic reticulum storage disorder with unique histopathologic findings characterized by abnormal accumulation of fibrinogen in the hepatocyte cytoplasm.^1^ In humans, fibrinogen‐positive cytoplasmic inclusions may be present in a variety of clinical conditions including chronic hepatitis B infection, chronic liver disease,^2^ alpha‐1‐antitrypsin deficiency (OMIM 107400) associated with disease‐causing variants in *SERPINA1*,^3^, ^4^, ^5^ and hypofibrinogenemia with hepatic storage (OMIM 202400) associated with disease‐causing variants in *FGA*,^6^ *FGB*,^7^ and *FGG*.^8^ Fibrinogen‐positive cytoplasmic inclusions can be classified into 3 groups according to their morphologic and ultrastructural appearance.^9^ Type I is characterized by polygonal inclusions of irregular outline that appear as dilated cisternae of the rough endoplasmic reticulum, filled with densely packed tubular structures, arranged in fingerprint‐like curved bundles on electron microscopy. Type II, on the other hand, has a ground‐glass appearance with ultrastructural features of granular or fibrillar material accumulated in dilated cisternae.^9^ In type III, round eosinophilic globules surrounded by a clear halo are observed. Ultrastructurally, these globules are composed of central tubular structures. All types of inclusions are negative or weakly positive for the periodic acid‐Schiff (PAS) reaction and for PAS with diastase (DPAS), making the diagnosis almost pathognomonic.

In cattle, the occurrence of large ground‐glass cytoplasmic inclusions in the hepatocytes of 6 Japanese black calves was reported and HFSD was diagnosed.^10^ However, the underlying etiology of the disease remained unclear. Our aim was to describe the clinical and histological phenotype observed in a Wagyu calf affected by HFSD, and to identify the etiology using a trio whole‐genome sequencing approach.

## 2 CASE DESCRIPTION

### 2.1 Clinical and histological appearance

A 5‐month‐old male Wagyu calf, weighing 79 kg, was admitted to the Clinic for Cattle of University of Veterinary Medicine Hannover, with a history of recurrent respiratory disease. The calf was born naturally, and was vital and alert after birth. Growth rate and development in the first few weeks of life were reported to be below expectations. A first episode of illness with decreased appetite and obtundation was observed at 4 months of age. The attending veterinarian suspected respiratory disease, which was treated with oxytetracyline (10 mg/kg IM once daily for 3 days) and non‐steroidal anti‐inflammatory drugs (meloxicam, 0.5 mg/kg SC). Because of lack of treatment response the calf was switched to gamithromycin (6 mg/kg SC as a single dose) and later to procaine penicillin (10 mg/kg IM daily for 3 days) and received a single dose of dexamethasone (0.02 mg/kg IM). The calf was referred to the clinic for diagnostic evaluation because of unsatisfactory response to previous treatment.

On admission, the calf appeared lethargic. The coat was dull and shaggy. Pulse and respiratory rate were 84 beats/min and 20 respirations/min, respectively; rectal temperature was 38.6°C. The calf appeared to be slightly dehydrated. The mucous membranes were pale, and serous nasal discharge was present from both nostrils. Clinical examination of the respiratory and cardiovascular systems identified no abnormalities. The rumen was poorly filled, without stratification and without any motility. The rectum contained small amounts of dark green dry feces partially coated with sticky mucus. Thoracic ultrasonography showed no abnormalities of the interpleural space or lung surface. The rumen appeared empty, and the abomasum and small intestinal loops showed only minimal motility with little content. Arterial blood gas analysis disclosed a marked metabolic acidosis, primarily caused by increased L‐lactate and total protein concentrations, consistent with a combined strong ion and *A*_tot_ acidosis. Hematology indicated moderate leukocytosis, increased erythrocyte count, and increased PCV. These results, in combination with the clinical examination findings, were attributed to dehydration at the time of admission. Blood biochemistry results showed markedly increased plasma urea and creatinine concentrations, and increased activities of aspartate aminotransferase (AST), glutamate dehydrogenase (GLDH), and creatine kinase (CK). Plasma cholesterol concentration was decreased (Table S1). Based on the clinical findings and blood biochemistry, severe dehydration and acidemia were considered the primary metabolic disturbances to be addressed therapeutically. Intravenous fluid therapy with 5 L of isotonic saline and 500 mL of 8.4% NaHCO_3_ solution was administered over a period of 6 hours. Venous blood gas analysis repeated after completion of this initial treatment showed that the acidemia and hyperlactatemia were corrected (Table S1). At this time, the PCV remained slightly above the upper reference limit, whereas the total plasma protein concentration had decreased below the reference range. Isotonic saline (5 L) was administered IV over the next 10 hours because blood urea nitrogen and creatinine concentrations remained markedly increased.

Blood testing performed on the next day, after completion of additional fluid therapy, disclosed a leukocyte count within the reference range and slightly increased red blood cells count (RBC) and PCV. Compared with the time of admission, total bilirubin concentration and activities of AST and GLDH were increased, whereas activities of CK and GGT were decreased. With fluid therapy, plasma total protein and plasma potassium concentrations decreased. Only a moderate decrease in blood urea nitrogen and plasma creatinine concentrations was observed compared with results obtained at the time of admission. To treat persistent azotemia, 10 L of isotonic saline were administered over 12 hours and 20 g of KCl were given PO. Another 5 L of isotonic saline was given IV over 12 hours because plasma creatinine concentration remained increased. On the second day of treatment, the calf was bright, alert, and responsive with normal appetite and thirst. Plasma urea and creatinine concentrations and activity of CK were decreased by approximately 50%, but marked increases in plasma AST, GGT, and GLDH activity were observed. The calf continued to receive 5 L of isotonic saline IV for another 24 hours.

Renal creatinine clearance and fractional excretion of sodium were calculated from spontaneously voided urine and a blood sample, both collected on day 5 and > 48 hours after cessation of IV fluid therapy and were found to be 1.8 mL/min and 0.54%, respectively. Urinary gamma‐glutamyl transferase (GGT) activity was 6 U/mL, specific gravity was 1.010 and a pH was 6.5. Urinary GGT activity was below the detection limit. Liver enzyme activities continued to increase, whereas plasma total bilirubin decreased to concentrations within the reference range.

**FIGURE 1:**
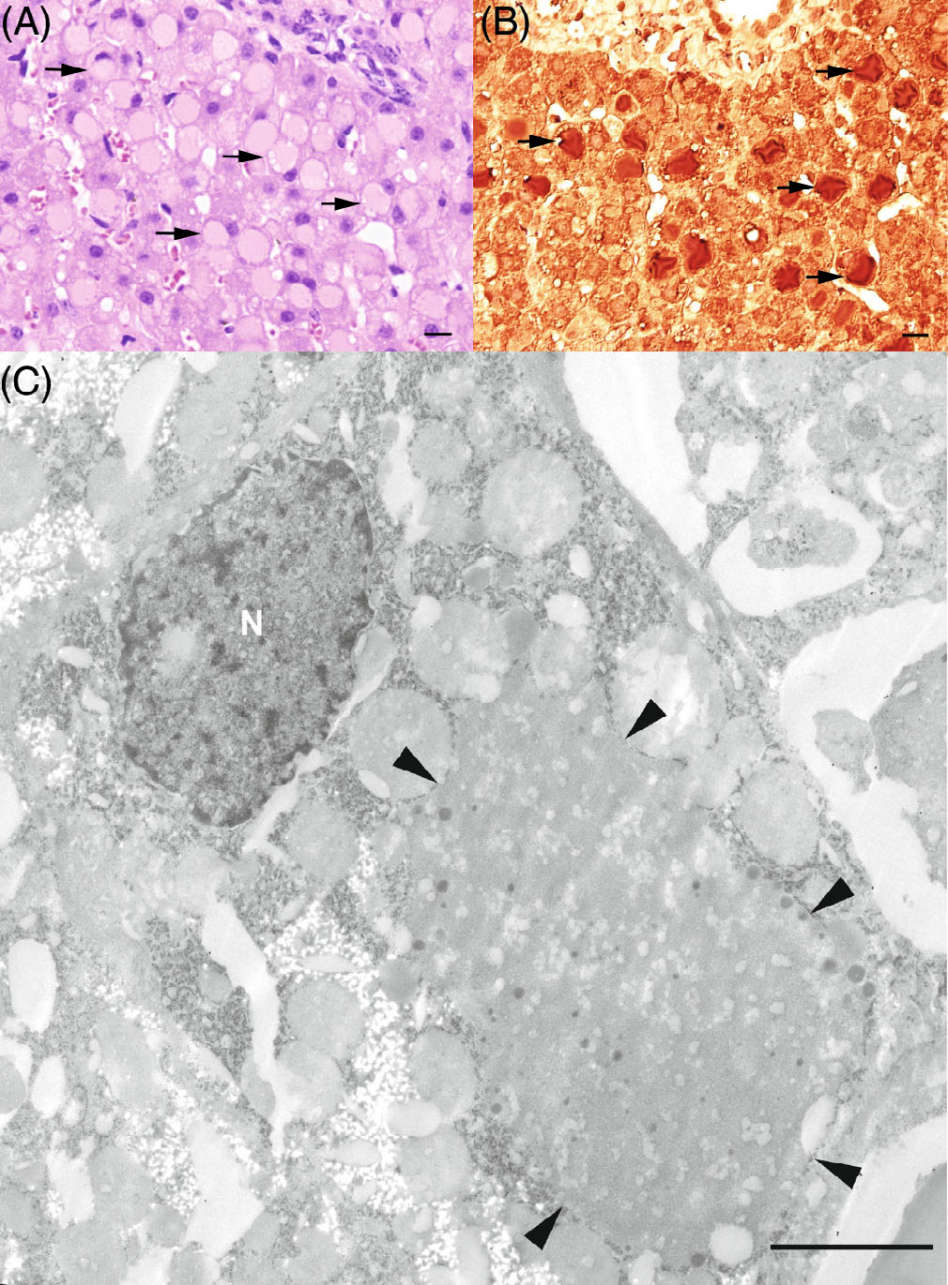
Hepatic histologic findings in the HFSD‐affected Wagyu calf. (A) the liver shows a lobular architecture with regular hepatocytic cords. Disseminated multiple hepatocytes are moderately swollen and mostly show a single, sharply demarcated faintly eosinophilic cytoplasmic inclusion with ground glass appearance with the nucleus displaced to an eccentric position (arrow); ×40 HE. (B) Cytoplasmic vacuoles in the hepatocytes intensely stained with a rabbit polyclonal antibody to fibrinogen with microwave pretreatment in citrate buffer, pH 6.0, a goat anti‐rabbit‐biotin antibody and the avidin‐biotin peroxidase complex (ABC) method; ×40. (C) Electron microscopy of the hepatocytes showing membrane‐bound cytoplasmic inclusions filled with fine‐grained material interspersed with a few coarse‐grained electron‐dense granules; arrowheads are denoting the inclusions. Bar = 2.5 μm.

Because the underlying cause of the increased liver enzyme activities and the patient's illness remained unclear, a liver biopsy was performed, and the tissue specimens were fixed in 10% buffered formalin. Histologically, disseminated multiple hepatocytes were moderately swollen and mostly showed a single, sharply demarcated cytoplasmic, slightly eosinophilic inclusion with a ground‐glass appearance, with the nucleus displaced to an eccentric position (Figure 1A). Occasionally, small, almost transparent vacuoles were present and single‐cell necrosis was seen. The cytoplasmic inclusions did not stain with the periodic acid‐Schiff (PAS)‐reaction and PAS with diastase (DPAS). Using a rabbit polyclonal antibody against fibrinogen (working dilution 1:500 in phosphate‐buffered saline [PBS]; Dako, Germany) with microwave pretreatment in citrate buffer, pH 6.0, a goat anti‐rabbit‐biotin antibody, and the avidin‐biotin peroxidase complex (ABC) method, the cytoplasmic vacuoles in the hepatocytes stained intensely (Figure 1B). Using the pop‐off‐technique,^11^ electron microscopy disclosed hepatocytes with membrane‐bound cytoplasmic inclusions filled with fine‐granular material admixed with a few coarse‐grained electron‐dense granules (Figure 1C). No fingerprint tubular material was observed.

The animal remained in the hospital 19 days to monitor recovery, and a steady decrease in liver enzyme activities was observed. The calf was discharged after 19 days. Based on the clinical and histopathologic findings, a form of hepatic fibrinogen storage disease (HFSD) was diagnosed. A follow‐up discussion with the attending veterinarian indicated that the animal had fully recovered and was still on the farm. All blood results are summarized in Table S1.

### 2.2 Genetic analysis

**FIGURE 2:**
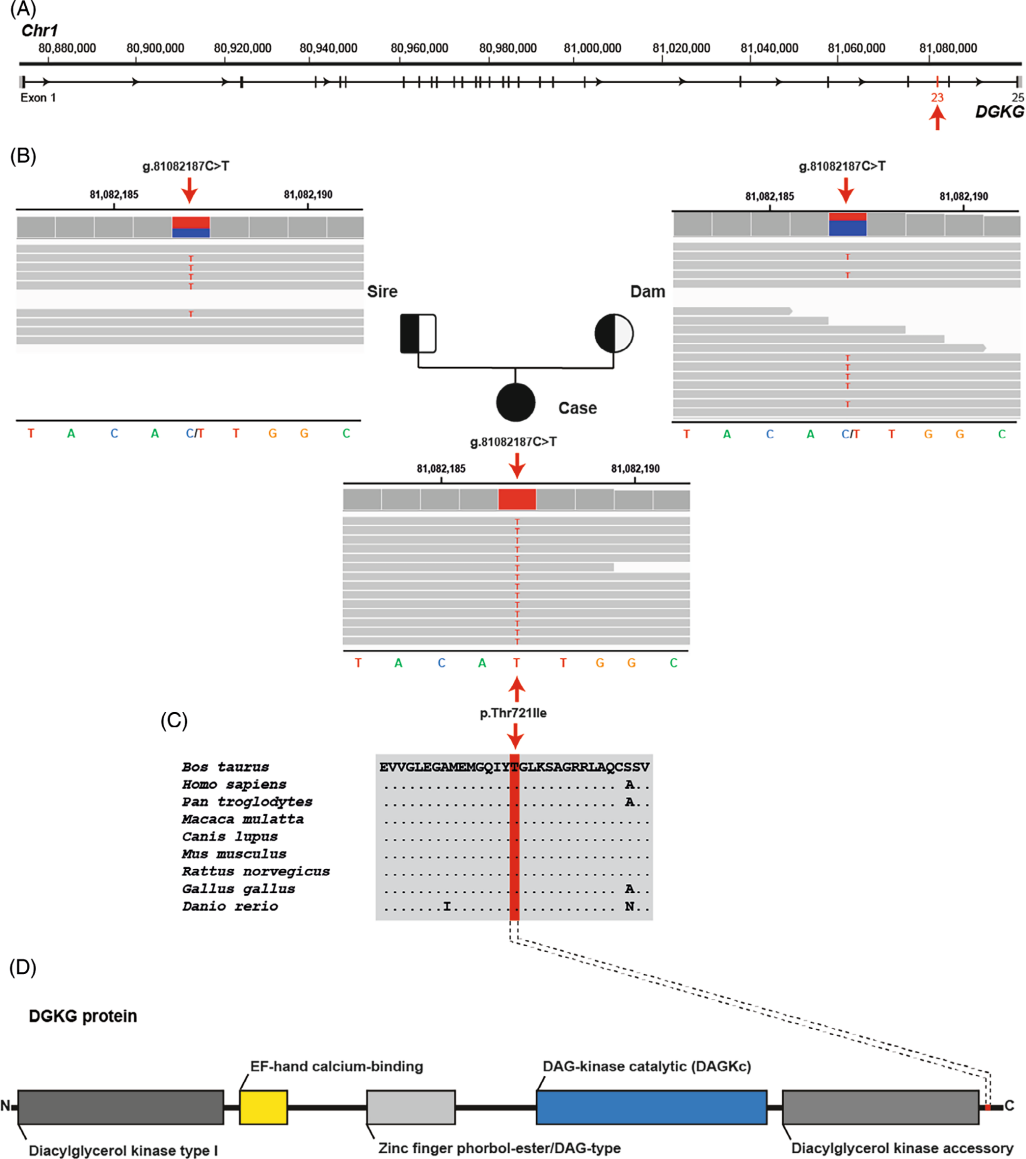
A homozygous *DGKG* missense variant in the HFSD‐affected Wagyu calf. (A) *DGKG* gene structure showing the location of the variant on chromosome 1, exon 23 (red arrow). References to the bovine *DGKG* gene correspond to the NCBI accessions NC_037328.1 (chromosome 1, ARS‐UCD1.3), XM_002684869.5 (bovine *DGKG* mRNA). (B) IGV screenshot showing the Chr1: g. 81082187C>T variant homozygous in the affected calf (shown below) and heterozygous in both parents (top left: sire; top right: dam) revealed by whole‐genome sequencing. (C) Multiple sequence alignment of the DGKG protein encompassing the region of the p.Thr721Ile variant reveals complete evolutionary conservation across species. Protein sequence accession numbers in NCBI for each species are XP_002684915.2 (*Bos taurus*), NP_001337.2 (*Homo sapiens*), XP_001152821.1 (*Pan troglodytes*), XP_001092912.1 (*Macaca mulatta*), XP_545239.2 (*Canis lupus*), NP_619591.1 (*Mus musculus*), NP_037258.1 (*Rattus norvegicus*), XP_422650.4 (*Gallus gallus*), XP_696120.6 (*Danio rerio*). (D) Schematic representation of the bovine DGKG protein and its functional domains obtained from the InterPro database (https://www.ebi.ac.uk/interpro/ accession number: A0A3Q1LSM5). The position of the p.Thr721Ile variant is indicated in red.

We evaluated the hypothesis of a congenital disorder using whole‐genome sequencing (WGS; see Data S1 for details of WGS analysis).^12^, ^13^, ^14^ Both the dam and sire of the affected calf had no clinically apparent abnormalities. Therefore, it was difficult to predict whether the disorder was dominant or recessive, so we hypothesized 2 different possible scenarios: a spontaneous, fully penetrant dominant‐acting *de novo* mutation and a recessive mutation that was present in the homozygous state and inherited from both parents. Assuming recessive inheritance in a trio‐based approach, filtering the WGS data for homozygous coding variants present in the calf and heterozygous in the parental genomes identified 22 017 variants, of which 81 were protein‐changing with a predicted high or moderate effect (Table 1). These 81 variants were further screened for their occurrence in a global control cohort of 4540 genomes from a variety of breeds, identifying 10 remaining protein‐changing variants that were exclusively homozygous in the genome of the affected calf and heterozygous in its parents (Tables 1 and S2). Tissue expression of RNA in humans and cattle using publicly available databases (https://www.ncbi.nlm.nih.gov and https://ianimal.pro) for the 9 genes affected by these 10 missense variants showed that they were all widely expressed in different tissues. Seven variants of uncertain importance were classified as neutral (Table 2), whereas 3 missense variants were potentially deleterious according to in silico predictions based on sequence homology using the Protein Variation Effect Analyzer (PROVEAN) web server (https://pubmed.ncbi.nlm.nih.gov/25851949/). Of these 10 remaining private variants, a single variant affected a putative candidate gene for the observed liver phenotype (Figure 2A; Table 2). This homozygous variant at chr1: 81082187C>T represents a missense variant in the *DGKG* gene, encoding the enzyme diacylglycerol kinase gamma involved in hepatic lipid metabolism (XM_002684869.5: c.2162C>T; Figure 2B). It alters the encoded amino acid of DGKG residue 721 and the threonine‐to‐isoleucine substitution (XP_002684915.3:p.Thr721Ile) affects an evolutionarily conserved amino acid (Figure 2C,D). The other 2 homozygous missense variants, which are predicted to be deleterious, affect genes with no known role in liver storage diseases. These are *TIAM2*, which encodes a Rac1‐specific guanine nucleotide exchange factor that, when dysregulated, is associated with diseases in humans including cancer, immunological and neurological disorders,^15^ and *PKD1*, which is known to be associated with dominant inherited polycystic kidney disease, which is characterized primarily by kidney cysts and may be accompanied by liver cysts (OMIM 601313). The number of heterozygous carriers in the global control cohort for these 2 variants was higher than the 3 heterozygous *DGKG* carriers observed in the Wagyu breed alone (Table 3). Furthermore, the occurrence does not seem to be restricted to the Wagyu breed. For *TIAM2* there are Angus cattle, and for *PKD1* there are Buryat and Hanwoo cattle carrying the variant alleles in addition to Wagyu cattle (Table 3).

**TABLE 1:** Results of the variant filtering of the HFSD‐affected calf using the whole‐genome sequence data from both parents and from 5483 control genomes.

Filtering step	Homozygous variants	Heterozygous variants
All variants in the affected calf	3 339 131	4 401 451
Private variants in the affected calf	27 179	3797
Private variants in the affected calf with obligatory carrier parents (protein‐changing)	22 017 (81)	NA
Protein‐changing private variants with obligatory carrier parents (recessive inheritance) after comparison with a global cohort	10	NA
Protein‐changing private variants absent in both parents (de novo mutations)	NA	0

Abbreviation: NA, not applicable.

**TABLE 2:** Pathogenicity prediction results for the 10 homozygous protein‐changing variants present exclusively in the genome of the HFSD‐affected calf and absent from the global control cohort of 5483 genomes from a variety of breeds.

Gene	OMIM	Gene function/associated disorder in humans	Protein change	Number of heterozygous carriers	Breed occurrence	Predicted effect^a^
*DGKG*	601854	Phosphatidic acid functions both in signaling and phospholipid synthesis.	p.Thr721Ile	3	Wagyu	Deleterious
*LRP1*	107770	Keratosis pilaris atrophicans	p.Pro4193Leu	3	Wagyu	Neutral
*TYRP1*	115501	Albinism, oculocutaneous, type III	p.Arg471Trp	18	Wagyu, Hanwoo, Japanese Native, Yanbian	Neutral
*EPB41L2*	603237	Adaptor in linking transmembrane proteins to the cytoskeleton	p.Ala600Val	10	Wagyu	Neutral
*TIAM2*	604709	Involved in neural cell development	p.Arg547His	5	Wagyu, Angus	Deleterious
*TMEM97*	612912	Plays a role in controlling cellular cholesterol levels	p.Ile102Met	17	Wagyu, Altai, Menggu, Hanwoo, Kazakh	Neutral
*RTTN*	610436	Microcephaly, short stature, and polymicrogyria with seizures	p.Ile2146Phe	5	Wagyu, Japanese Native	Neutral
*PKD1*	601313	Polycystic kidney disease 1	p.Ala3769Gly	15	Wagyu, Buryat, Hanwoo	Neutral
			p.Met4084Lys	17	Wagyu, Buryat, Hanwoo	Deleterious
*ZNF205*	603436	May be involved in transcriptional regulation	p.Pro56Leu	7	Wagyu, Hanwoo, Japanese Native	Neutral

Abbreviations: N, number; OMIM, Online Mendelian Inheritance in Man (https://omim.org/).

^a^ Based on PROVEAN score.

**TABLE 3:** Association of the missense variant in *DGKG* with the HFSD phenotype in Wagyu cattle.

	*DGKG* genotype		
	CC (*FSF*)	CT (*FSC*)	TT (*FSS*)
HFSD‐affected calf			1
Obligate carriers^a^		2	
Other Wagyu cattle^b^	152	21	1
Sequenced Wagyu cattle^c^	29	3	
Sequenced control cattle from various breeds^c^	5451		
Total (Wagyu)	5632 (181)	26 (26)	2 (2)

Abbreviations: *FSC*, tested carrier of HFSD (heterozygous); *FSF*, tested non‐carrier/free of HFSD; *FSS*, tested true carrier of HFSD (homozygous affected).

^a^ Parents of affected animals were classified as obligate carriers.

^b^ Phenotypes are unknown.

^c^ Swiss Comparative Bovine Resequencing project and 1000 Bull Genomes project with 32 Wagyu cattle.

Variant filtering identified no private heterozygous protein‐changing variants present in the genome of the HFSD‐affected calf and absent in both parental genomes and in 5483 controls (Table 1). Thus, a pathogenic *de novo* mutation that could have occurred in a single parental gamete or during early embryonic development of the calf seems unlikely in the affected animal.

## 3 DISCUSSION

In our study, a comprehensive clinical, histologic, and genetic evaluation of a Wagyu calf with HFSD was performed. Based on the hepatic histologic findings, it is reasonable to assume that the HFSD‐affected calf had fibrinogen‐positive cytoplasmic inclusions similar to human type II.^9^ Similar findings were previously reported in 6 HFSD‐affected Japanese Black calves, 4 of which also had respiratory disease.^10^ Histological examination of liver tissue from affected animals disclosed hepatocytes with large cytoplasmic inclusions, morphologically characterized by a uniform, homogeneous, glassy appearance. Similar to our study, PAS staining was negative, whereas immunohistochemistry for fibrinogen showed intense positivity.^10^ However, the etiology of the disease was not investigated in this earlier study. Four different breeds are known collectively as Wagyu cattle: Japanese Black, Japanese Brown, Japanese Shorthorn, and Japanese Polled. The latter 2 breeds have stronger genetic influences from European breeds, whereas Japanese Black forms a distinct group from the Asian cattle group, which includes Korean cattle and Japanese Brown.^16^ Therefore, it could be speculated that the previously reported Japanese Black calves affected by HFSD could be caused by the *DGKG* variant described here, which was found in a Wagyu calf reared in Europe.

Our study suggests a possible genetic, most likely recessive, cause of HFSD in Wagyu cattle. The trio‐based WGS approach identified 10 protein‐changing variants that were exclusively homozygous in the genome of the affected calf, consistent with monogenic recessive Mendelian inheritance. After gene function analysis, taking into account the occurrence of the variant alleles in a global control cohort, the rarity and breed specificity of the variant, and *in silico* effect predictions, only the homozygous variant affecting *DGKG* was considered the most likely genetic cause of the observed phenotype. Diacylglycerol kinase gamma protein (DGKG) is a member of the diacylglycerol kinase (DGK) enzyme family, which comprises 10 isozymes in mammalian species.^17^, ^18^ It converts diacylglycerol to phosphatidic acid and regulates the respective concentrations of these 2 bioactive lipids.^19^ Diacylglycerol is a neutral lipid that plays an important role as a second messenger, activating the convectional and novel protein kinase C family, RasGRP, Unc‐13, and canonical transient receptor potential channels.^18^, ^20^ Phosphatidic acid controls several signaling proteins such as phosphatidylinositol‐4‐phosphate 5‐kinase, Ras GTPase‐activating protein, C‐Raf and atypical protein kinase C.^17^, ^18^, ^21^ In addition, DGKG is involved in a wide range of cellular trafficking between the endoplasmic reticulum and the Golgi apparatus^22^ and is moderately expressed in liver.^23^ Unfortunately, to carry out functional follow‐up studies, suitable tissue samples were not available to perform RNA or protein studies, so we could not assess the effect of the variant on the transcript, encoded protein or both.

In humans, forms of HFSD such as hypofibrinogenemia and alpha‐1‐antitrypsin deficiency may have a genetic etiology. Histologically, 3 different morphologic types of fibrinogen inclusions are classified based on their morphologic and ultrastructural features (Type I‐III).^9^, ^24^ In our case, fibrinogen‐positive cytoplasmic inclusions resembling type II were detected, but no variants in known candidate genes for HFSD were identified. In the presence of type II/III inclusions, humans are diagnosed with HFSD, which was previously thought to consist exclusively of fibrinogen.^9^, ^25^ However, these inclusions appear to contain multiple proteins such as C4d and C‐reactive protein^1^ and therefore the dysfunction of proteins other than the fibrinogen family might be expected.

In human medicine, the trio‐based WGS approach is being used as a diagnostic tool for monogenic disorders mostly in pediatrics including the detection of single nucleoid variants/indels, uniparental disomy, imprinted genes, short tandem repeat expansions, and mitochondrial variants.^26^, ^27^ Moreover, trio‐WGS, when used by primary care physicians in clinical settings with a high risk of rare pediatric conditions, provides an effective single‐pass screening test for children.^25^

We were able to identify a plausible, highly probable pathogenic candidate variant in *DGKG* as the cause of a recessive form of HFSD in cattle. This report serves to alert Wagyu breeders to the possible occurrence of congenital HFSD and will allow genetic testing to prevent the unintended occurrence of more affected cattle. Our case report illustrates the utility of precision diagnostics, including genomics, in understanding rare diseases and the value of monitoring cattle breeding populations for deleterious genetic diseases. Future studies evaluating the functionality of the DGKG enzyme in the presence of the missense variant will be valuable in understanding the biological impact of the mutation.

## CONFLICT OF INTEREST DECLARATION

Michael Karl, Michael Pohlers, and Lutz Plobner are employees of Agrobiogen, a veterinary diagnostic laboratory that provides genetic testing for animals, including Wagyu cattle. No other authors declare a conflict of interest.

## OFF‐LABEL ANTIMICROBIAL DECLARATION

Authors declare no off‐label use of antimicrobials.

## INSTITUTIONAL ANIMAL CARE AND USE COMMITTEE (IACUC) OR OTHER APPROVAL DECLARATION

Authors declare no IACUC or other approval was needed.

## HUMAN ETHICS APPROVAL DECLARATION

Authors declare human ethics approval was not needed for this study.

## Supporting information


**Data S1.** Supporting Information.

**Table S1.** Results of the blood analysis of the HFSD‐affected calf.

**Table S2.** List of the remaining variants after the comparison with the global control cohort of 5483 control genomes from other breeds and after IGV visual inspection, revealing 10 homozygous protein‐changing variants with a predicted moderate effect present only in the HFSD‐affected calf.
