# Supplementary material for: A missense variant in DGKG as a recessive functional variant for hepatic fibrinogen storage disease in Wagyu cattle
Source: J Vet Intern Med. 2023 Sep 8;37(6):2631–7. doi: 10.1111/jvim.16865 (PMC10658517; doi:10.1111/jvim.16865)
Supplement: Supplementary file 1 — Data S1. Supporting Information. [file JVIM-37-2631-s002.pdf]

Genomic Analysis Details

WGS using the Illumina NovaSeq6000 was performed on DNA extracted from EDTA-blood of the HFSD-affected calf, its dam, and from semen from its sire. The sequenced reads were mapped to the ARS-UCD1.2[12] reference genome, resulting in an average read depth of approximately 18× in the calf, 11× in the dam, and 19× in the sire, and then and then processed as previously reported. [13] To find private variants, the genotype of the affected calf was compared to 5483 controls, including 943 cattle genomes from different breeds sequenced as part of the ongoing Swiss Comparative Bovine Resequencing Project and 4540 genomes from different breeds, including 32 Wagyu cattle, included in Run 9 of the 1000 Bull Genomes Project.[12] The Integrative Genomics Viewer (IGV)[14] version 2.0 software was used for visual inspection of genomic regions containing candidate genes (*SERPINA1*, *FGA6*, *FGB7*, and *FGG*) and the identified functional variants. No functional variants were identified in the above-mentioned candidate genes.
