# Supplementary material for: A missense variant in DGKG as a recessive functional variant for hepatic fibrinogen storage disease in Wagyu cattle
Source: J Vet Intern Med. 2023 Sep 8;37(6):2631–7. doi: 10.1111/jvim.16865 (PMC10658517; doi:10.1111/jvim.16865)
Supplement: Supplementary file 2 — Table S1. Results of the blood analysis of the HFSD‐affected calf. [file JVIM-37-2631-s001.pdf]

**Table S1.** Results of the blood analysis of the HFSD-affected calf**.**

| **Parameter** | **Reference** | **Sampling time** | | | | | | | | | | | |
|---|---|---|---|---|---|---|---|---|---|---|---|---|---|
| | | **d0** | **d0+6h** | **d1** | **d1+12h** | **d2** | **d3** | **d4** | **d5** | **d7** | **d12** | **d19** | **m16** |
| WBC | 8,000-10,000/ µL | 14,000 | - | 7.700 | - | 5,400 | - | - | 19,400 | 6,200 | 7,600 | 5,100 | 6,300 |
| RBC | 6,000-8,000 * $10^6$/ µL | 14,70 | - | 11.80 | - | 11,60 | - | - | 11,60 | 11,70 | 10,10 | 9,71 | 6,35 |
| PCV | 25.0-35.0% | 49.7 | - | 39.8 | - | 39.0 | - | - | 38.3 | 38.2 | 37.1 | 32.9 | 32.2 |
| Total bilirubin | < 7,0 µmol/L | 6.3 | - | 30 | - | 31.1 | - | - | 7.5 | 13.5 | 4.3 | 6.3 | 10.4 |
| AST | < 100 U/L | 249 | - | 430 | - | 509 | - | - | 550 | 237 | 104 | 67 | 65 |
| GGT | <33 U/L | 30 | - | 21 | - | 31 | - | - | 42 | 52 | 33 | 25 | 16 |
| GLDH | < 14 U/L | 47.3 | - | 101 | - | 221 | - | - | 580 | 156 | 58.2 | 54.9 | 10.4 |
| Cholesterol | > 3.0 µmol/L | 0.57 | - | 0.74 | - | 0.75 | - | - | 0.72 | 0.64 | 0.62 | 0.75 | 1.86 |
| CK | <350 U/L | 3049 | - | 1442 | - | 642 | - | - | 94 | 93 | - | - | 53 |
| Total protein | 60.0-80.0 g/L | 77 | 59 | 62 | 62 | 66 | 68 | 75 | 72 | 74 | 70 | 70 | 64 |
| Urea | <9.0 mmol/L | 32.7 | - | 20.3 | - | 8.2 | - | - | 6.94 | 6.88 | 5.58 | 3.08 | 2.51 |
| Creatinine | <150 µmol/L | 490 | 401 | 316 | 208 | 182 | 174 | 195 | 141 | 122 | 104 | 115 | 165 |
| Albumin | 30.0-40.0 g/L | 35.7 | - | 30.6 | - | 31.1 | - | - | 33.8 | 32.7 | 29.1 | 30.2 | 29.9 |
| Ca | 2.1-3.0 mmol/L | 2.25 | - | 2.20 | - | 2.14 | - | - | 2.13 | 2.23 | 2.36 | 2.59 | 2.3 |
| Mg | 0.7-1.2 mmol/L | 0.93 | - | 0.74 | - | 0.52 | - | - | 0.43 | 0.51 | 0.71 | 1.00 | 0.77 |
| P | 1.1-2.4 mmol/L | 1.65 | - | 1.76 | - | 1.87 | - | - | 1.78 | 1.97 | 2.06 | 2.35 | 1.81 |
| Na | 135-145 mmol/L | 134 | 145 | 142 | 146 | 140 | 142 | 136 | 141 | 135 | 140 | 138 | 135 |
| K | 3.4-4.5 mmol/L | 2.8 | 2.0 | 2.1 | 2.3 | 3.26 | 3.1 | 2.5 | 3.69 | 3.60 | 3.89 | 4.0 | 4,26 |
| Cl | 90-110 mmol/L | 106 | 109 | 104 | 100 | 98 | 111 | 103 | 98 | 96 | 98 | 94 | 99 |
| pH | | 7.174* | 7.326 | 7.387 | 7.324 | - | 7.328 | 7.340 | - | 7.346 | - | - | - |
| $pCO_2$ | mmHg | 34.9* | 50.6 | 42.2 | 44.1 | - | 40.3 | 40.9 | - | 51.9 | - | - | - |
| $pO_2$ | mmHg | 98.0* | 42.5 | 41.4 | 50.8 | - | 37.0 | 46.9 | - | 32.6 | - | - | - |
| $HCO_3$act | mmol/L | 12.6*+ | 26.0 | 25.2 | 22.6 | - | 20.8 | 21.6 | - | 27.8 | - | - | - |
| BE | mEq/L | -15.5* | 0.4 | 0.4 | -3.2 | - | -4.8 | -3.7 | - | 2.6 | - | - | - |
| $SO_2$sat | % | 94.5* | 68.6 | 74.3 | 79.5 | - | 61.4 | 75.2 | - | 52.2 | - | - | - |
| L-Lactate | mmol/L | 7.34* | 1.40 | 0.79 | 0.66 | - | 0.85 | 1.14 | - | 0.47 | - | - | - |

*, arterial; -, not available; d, day; h, hour; m, month; WBC, white blood cell; RBC, red blood cell; AST, aspartate aminotransferase; GGT, gamma-glutamyl transferase; GLDH, glutamate dehydrogenase; CK, creatinine kinase; Ca, calcium; Mg, magnesium; P, inorganic phosphorus; Na, sodium; K, potassium; Cl, chloride; $pCO_2$, partial pressure of carbon dioxide; $pO_2$, partial pressure of oxigen; $HCO_3$act, actual bicarbonate.
