# Supplementary material for: A missense variant in DGKG as a recessive functional variant for hepatic fibrinogen storage disease in Wagyu cattle
Source: J Vet Intern Med. 2023 Sep 8;37(6):2631–7. doi: 10.1111/jvim.16865 (PMC10658517; doi:10.1111/jvim.16865)
Supplement: Supplementary file 3 — Table S2. List of the remaining variants after the comparison with the global control cohort of 5483 control genomes from other breeds and after IGV visual inspection, revealing 10 homozygous protein‐changing variants with a predicted moderate effect present only in the HFSD‐affected calf. [file JVIM-37-2631-s003.pdf]

Table S1 - List of the remaining variants after the comparison with the global control cohort of 5483 control genomes from other breeds and after IGV visual inspection, revealing ten homozygous protein-changing variants with a predicted moderate effect present only in the HFSD-affected calf.

#CHROM	POS	REF	ALT	EFFECT	IMPACT	GENE	GENEID	FEATURE	FEATUREID	BIOTYPE	RANK	HGVS_C	HGVS_P
1	81082187	C	T	missense_variant	MODERATE	DGKG	518523	transcript	XM_002684869.5	protein_coding	23	c.2162C>T	p.Thr721Ile
5	56232954	G	A	missense_variant	MODERATE	LRP1	533894	transcript	XM_024992496.1	protein_coding	81	c.12578C>T	p.Pro4193Leu
8	31632362	G	A	missense_variant&splice_region_variant	MODERATE	TYRP1	282105	transcript	NM_174480.3	protein_coding	8	c.1411C>T	p.Arg471Trp
9	68978040	G	A	missense_variant&splice_region_variant	MODERATE	EPB41L2	538959	transcript	XM_010808553.3	protein_coding	13	c.1799C>T	p.Ala600Val
9	91763887	G	A	missense_variant	MODERATE	TIAM2	100139161	transcript	XM_015472916.2	protein_coding	6	c.1640G>A	p.Arg547His
19	19813579	C	G	missense_variant	MODERATE	TMEM97	511378	transcript	NM_001034403.1	protein_coding	3	c.306C>G	p.Ile102Met
24	7236837	A	T	missense_variant	MODERATE	RTTN	615378	transcript	NM_001206207.1	protein_coding	47	c.6436A>T	p.Ile2146Phe
25	1627971	A	T	missense_variant	MODERATE	PKD1	504986	transcript	XM_003587798.5	protein_coding	45	c.12251T>A	p.Met4084Lys
25	1629426	G	C	missense_variant	MODERATE	PKD1	504986	transcript	XM_003587798.5	protein_coding	40	c.11306C>G	p.Ala3769Gly
25	2565879	C	T	missense_variant	MODERATE	ZNF205	514765	transcript	NM_001034473.2	protein_coding	3	c.167C>T	p.Pro56Leu
